# Supplementary material for: Non-oncological gynecological diagnoses in a women’s health care service during the pandemic caused by the Severe Acute Respiratory Syndrome Coronavirus 2
Source: PLoS One. 2023 Mar 23;18(3):e0282039. doi: 10.1371/journal.pone.0282039 (PMC10035870; doi:10.1371/journal.pone.0282039)
Supplement: Attachment — Submitted filename: Review_PlosOne.docx [file pone.0282039.s001.docx]

Regarding

Non-oncological gynecological diagnoses in a women’s health care service during the pandemic caused by the Severe Acute Respiratory Syndrome Coronavirus 2 (SARSCoV- 2).

Manuscript Number: PONE-D-22-10074

I would like to thank you for the opportunity to review this manuscript. The study “Non-oncological gynecological diagnoses in a women’s health care service during the pandemic caused by the Severe Acute Respiratory Syndrome Coronavirus 2 (SARSCoV-2)” addresses an interesting and new topic aiming at analyze the sociodemographic and clinical factors and non-oncological gynecological diagnoses before and during the initial months of the COVID-19 pandemic in women that were assessed in a tertiary reference outpatient clinic. However, there are some issues that before publication should be solved. Please, find below my comments:

Comments

1. The abstract is good, it brings the main information of the manuscript.
2. The introduction is well written, but I cannot identify a clear hypothesis.
3. The objective is correctly worded: clear and succinct and with specialized terms suitable for a general journal
4. The materials and methods described are appropriate to achieve the proposed objectives; are described clearly, completely, and succinctly to answer the proposed question. As for the data collection process and the instruments used, they are clearly described, but according to STROBE Statement we should report:

- Inclusion criteria should be more detailed (women over 18 years of age?)
- Bias: Describe any efforts to address potential sources of bias
- Study size: Information on study size is separate. The calculation result is in the first sentence of the results section, it should be in the method.

5. Statistical analysis is adequate but according to STROBE Statement we should report “Explain how missing data were addressed”
6. Results are clear, appropriate, and correctly presented. The tables contain useful information and are arranged accordingly, but:

- Table 1 appears to be missing information. Just below "Age at first intercourse" there are several "yes" and "no", but it's not clear where they belong.
- Figure 1 is low resolution

7. It presents and discusses the points of convergence and divergence in relation to other authors. It mentions possible generalizations and/or practical applications or limitations from the results obtained.

Criteria for Publication – Plos One

1. The study presents the results of original research: Yes

2. Results reported have not been published elsewhere: Ok

3. Experiments, statistics, and other analyses are performed to a high technical standard and are described in sufficient detail: Yes

4. Conclusions are presented in an appropriate fashion and are supported by the data: Yes

5. The article is presented in an intelligible fashion and is written in standard English: I am not qualified to rate this question

6. The research meets all applicable standards for the ethics of experimentation and research integrity: Yes

7. The article adheres to appropriate reporting guidelines and community standards for data availability: There is no information on this issue
